# Surveying knowledge, practice and attitudes towards intervention fidelity within trials of complex healthcare interventions

**DOI:** 10.1186/s13063-018-2838-6

**Published:** 2018-09-19

**Authors:** Daragh McGee, Fabiana Lorencatto, Karen Matvienko-Sikar, Elaine Toomey

**Affiliations:** 1 School of Medicine, Clinical Science Institute, National University of Ireland Galway, University Road, Galway, Ireland H91 TK33; 2 Centre for Behaviour Change, University College London, 1-19 Torrington Place, London, WC1E 7HB UK; 3 School of Public Health, Western Gateway Building, University College Cork, Cork, Ireland T12 YN60; 4 Health Behaviour Change Research Group, Room 2058, School of Psychology, Arts Millennium Building, National University of Ireland Galway, University Road, Galway, Ireland H91 TK33

**Keywords:** Intervention fidelity, Complex interventions, Randomised control trials, Research methods, Survey

## Abstract

**Background:**

Intervention fidelity is the degree to which interventions have been implemented as intended by their developers. Assessing fidelity is crucial for accurate interpretation of intervention effectiveness, but fidelity is often poorly addressed within trials of complex healthcare interventions. The reasons for this are unclear, and information on the use of methods to enhance and assess fidelity in trials of complex interventions remains insufficient. This study aimed to explore the knowledge, practice and attitudes towards intervention fidelity amongst researchers, triallists and healthcare professionals involved with the design and conduct of trials of complex healthcare interventions.

**Methods:**

An online survey consisting of closed and open-ended questions exploring four sections (Demographics, Fidelity knowledge, Practice and Attitudes) was conducted. This was an opportunistic sample of individuals with experience of direct involvement in trials of complex healthcare interventions (e.g. design/development, conduct, evaluation).

**Results:**

Data from 264 participants representing 15 countries were analysed. The majority (65.9%, *n* = 174) of participants identified themselves as ‘Researchers’. The majority of participants were familiar with the term “intervention fidelity” (69.7%, *n* = 184) and indicated that fidelity is important (89.7%, *n* = 236). Mean self-reported understanding of fidelity was moderate. Although 68% (*n* = 182) had previously used strategies to assess (e.g. audio/video-recording sessions) and enhance (e.g. training manual) fidelity in trials of complex interventions, only a limited proportion of participants indicated always reporting these strategies in subsequent publications (30.9%, *n* = 56). Poor knowledge or understanding was the most commonly cited barrier to addressing intervention fidelity in trials (77.4%, *n* = 202). Over half of respondents (52.1%, *n* = 137) had never completed specific fidelity training or research, and the vast majority (89.7%, *n* = 236) would welcome specific training in this area.

**Conclusion:**

Despite good awareness of intervention fidelity and its importance, poor knowledge and understanding appears to be a key factor limiting how intervention fidelity is addressed in trials of complex interventions. Participants identified a need for further training and education in this area. Additionally, clarification of the terminology, definition and components of intervention fidelity would facilitate better understanding of the concept. A discrepancy between participants’ use of fidelity strategies and subsequent reporting raises concerns around inadequate fidelity reporting in the trials literature.

**Electronic supplementary material:**

The online version of this article (10.1186/s13063-018-2838-6) contains supplementary material, which is available to authorized users.

## Background

Trial evaluations typically focus on understanding whether or not interventions “work” to attain target outcomes, with comparatively less focus on understanding *how* and *why* interventions succeed or fail in attaining target outcomes. Complex interventions are interventions with several interacting components. This includes aspects such as the number and difficulty of behaviours involved, number of groups or organisational levels targeted by the intervention, number and variability of outcomes and the degree of flexibility or tailoring that the intervention permitted [1]. Given the greater capacity for variation in the implementation of these components and therefore greater scope for confounding variables to influence outcomes, the focus on understanding the *how* and *why* is of particular importance to trials of complex interventions [2, 3]. Intervention fidelity is the degree to which an intervention is implemented as intended in the original programme model or protocol by its developers [2–7], and is crucial for accurate interpretation of intervention outcomes. However, intervention fidelity is often poorly addressed within trials of complex healthcare interventions [4, 8–14]. The reasons for this are unclear, and information on the knowledge, practice and attitudes of stakeholders involved in trials of complex interventions towards methods that can be used to enhance and assess fidelity remains insufficient.

Intervention fidelity increases the internal validity of a trial such that the results are directly attributable to the intervention [3, 8]. For instance, if trials demonstrate non-significant results and did not measure fidelity, one cannot be sure that null results were due to an ineffective intervention or intervention components that were omitted. Similarly, if significant results were found and fidelity not measured, one cannot be sure if the results were due to an effective intervention or additional unintended components influencing the outcome [15]. This enables researchers to more accurately detect meaningful effects and establish causal relationships by reducing random and unintended variability. This enables a better understanding of how and why complex interventions have or have not worked and what the “active ingredients” of the intervention are [16, 17]. Fidelity strategies can be considered in terms of methods to enhance or improve fidelity of intervention implementation (e.g. treatment manuals), and methods to assess or monitor fidelity alongside trial outcome evaluations (e.g. provider self-report record) [8, 10, 14–18]. Reporting the intervention fidelity methods used in trials of complex interventions provides practitioners with adequate information to determine whether they are delivering the intervention as intended in real-life settings. This also enhances the external validity of a trial and increases reproducibility [8].

Despite the importance of intervention fidelity within complex interventions, numerous reviews have shown that the quality and scope of how fidelity has been enhanced and assessed across multiple areas is poor [4, 8–14]. For example, Walton et al. found that fewer than half of the included studies measured fidelity within complex health behaviour change interventions [13]. In a systematic review of fidelity methods used in complex behaviour change interventions promoting physical activity, Lambert et al. further identified a lack of attention to the quality of fidelity assessments, with few studies using objective methods [14]. Another review found that none of the 72 included articles reported either fidelity definitions or use of conceptual frameworks [4]. This is despite the availability of several conceptual frameworks and methodological guidance for assessing fidelity [9, 15]. However, existing frameworks differ in several respects. First, there is inconsistency and lack of agreement on terminology [4], with terms such as intervention fidelity, treatment fidelity, implementation fidelity and programme adherence often used interchangeably [4, 19, 20]. Furthermore, these frameworks differ in the specific dimensions and components that they argue constitute fidelity. Nonetheless, all frameworks agree that fidelity is a multidimensional concept, relevant at the intervention designer, provider and recipient level [11, 21–23]. The National Institutes of Health Behaviour Change Consortium (NIHBCC) fidelity framework attempted to synthesise this research, conceptualising fidelity as consisting of five domains [16]. These include; study design (a study’s intended hypotheses in relation to the underlying theory and mechanisms of action), training providers (referring to the providers’ ability to deliver the intervention as intended), delivery of treatment (providers’ actual delivery of the intended intervention) receipt of treatment (participants understand and are *able* to perform intervention skills and behaviours) and enactment (participants apply intervention skills and behaviours in real life) [16]. However, despite the comprehensiveness of the NIHBCC framework, debate remains about the addition of certain fidelity components such as treatment enactment [24] and many still view fidelity as only ‘the *delivery* of the intervention or treatment as intended’ [7, 25].

While existing reviews have documented limitations in how fidelity has been addressed within trials of complex interventions [4, 8–14, 23, 26], they have not investigated potential reasons underpinning the lack of investment into investigating fidelity. Perepletchikova et al. explored barriers to intervention fidelity amongst psychotherapists exclusively [27], and found lack of theory and specific guidelines, time, cost and labour to be strong barriers to implementing fidelity procedures [27]. Other barriers identified by their survey included lack of general knowledge and lack of editorial requirement. In a survey of school psychologists’ attitudes towards intervention fidelity in school-based interventions for children, Cochrane et al. found that although participants agreed fidelity was important, only 10.7% of psychologists reported always assessing it within individual interventions [28]. Reasons for this included time constraints and lack of understanding of and buy-in towards fidelity by teachers. However, there is no such information on the barriers and facilitators towards fidelity practices within trials of healthcare complex interventions, and the knowledge or attitudes amongst those involved in the design and conduct of these trials. Identification of such factors is essential to realise the potential contribution of fidelity data towards the interpretation, implementation and scalability of complex healthcare intervention trial findings.

This study therefore sought to explore the knowledge, practice (including barriers and facilitators to practice) and attitudes towards addressing intervention fidelity amongst researchers, triallists and healthcare professionals with experience of trials of complex healthcare interventions. It also sought to explore potential associations between self-reported fidelity knowledge/understanding and attitudes towards fidelity and (1) years of experience, (2) level of qualification and (3) research area.

## Methods

### Design

A cross-sectional web-based questionnaire study design was used.

### Target population

The target population was researchers, triallists and healthcare professionals with direct involvement in trials of complex healthcare interventions (e.g. design/development, conduct, evaluation) but excluding study subjects/patient participants. Such individuals could include academic researchers, research practitioners, clinicians, triallists, trial methodologists and statisticians from all areas of healthcare e.g. medicine, psychology, nursing/midwifery, allied health professionals. Participants were identified via professional groups, research networks and academic institutions. Complex interventions were defined as per the Medical Research Council (MRC) definition [1], as described previously. Participants with experience of pharmaceutical/drug trials only were not eligible to complete the survey as these were not considered “complex interventions” as defined by the MRC guidance [1].

### Questionnaire

A 34-item questionnaire (30 closed and 4 open-ended questions) was developed by DMc and ET (Additional file 1). Brief demographic and background information was collected from the participants, for example age, gender, country of work, area of healthcare research etc. No personal identifying information was collected. Survey questions were largely based on the NIHBCC conceptualisation of fidelity, but drawing on other prominent fidelity literature and previous fidelity questionnaires e.g. Carroll et al., Perepletchikova et al., Cochrane et al., Smith et al. [2, 3, 28, 29].

Questions were piloted for content, readability and feasibility of completion with seven researchers and research practitioners from a variety of research backgrounds including health services research, health psychology, trials methodology, implementation science and medical statistics. Feedback provided was mostly minor and related to clarifying certain aspects (e.g. providing a level of involvement for research practitioner as well as practitioner, providing an example of intervention theory/hypothesised mechanisms of action), or simplifying word use. Feedback from one researcher also resulted in the inclusion of an additional question to collect data on facilitators and barriers to fidelity. The final questionnaire was structured around three sections:

1. *Knowledge (5 questions)*
  The knowledge section asked participants whether they were familiar with the term “intervention fidelity”. Participants familiar with the term were shown statements describing components of intervention fidelity and asked to select which they felt were fidelity components. These statements were based on components as identified and defined previously by the NIHBCC (i.e. definitions of study design, provider training, treatment delivery, treatment receipt, treatment enactment) [15]. Two other components derived from existing fidelity literature [11] (“ensuring that training is given to providers as intended”, i.e. delivery of training and “ensuring adequate difference between the intervention and comparator groups”, i.e. treatment differentiation) were also shown. Participants could select more than one option or could add additional components. Participants who were not familiar with intervention fidelity were asked about familiarity with provided synonymous terms obtained from the literature [20]. Participants could select more than one term or add additional terms. All participants were asked to self-report their understanding of intervention fidelity on a scale from 1 (poor) to 10 (excellent).
2. *Practice and barriers and facilitators to practice (15 questions)*
  The practice section assessed participants’ previous use of specific fidelity strategies or fidelity frameworks and subsequent reporting (i.e. publishing/dissemination) of strategies used or results of fidelity assessments. This section also assessed the barriers and facilitators to these specific aspects. Participants were initially provided with the definition of intervention fidelity by Carroll et al. as previously detailed. Examples of specific strategies to enhance and assess fidelity such as the use of treatment manuals (enhance) or provider self-report record (assess) strategies as previously recorded in the fidelity literature were shown to participants [15, 16, 24, 30]. Participants were asked to identify specific methods they had previously used (if any) and could select more than one option or describe additional strategies or select a “none” option. Participants were also asked to rate frequency of use of assessment strategies, enhancement strategies and reporting of these strategies on a 5-point scale from 1 (never) to 5 (always). Participants were then asked if they had used specific validated tools or frameworks to inform how fidelity might be enhanced, assessed or reported (e.g. NIHBCC Treatment Fidelity Framework [9]). Participants could select more than one option or list additional frameworks or select a “none” option. Participants were then provided with a list of barriers (*n* = 14) and facilitators (*n* = 11) to addressing intervention fidelity previously identified in the literature [27, 28] (e.g. lack of journal requirement for publication), and asked to select which of these were barriers and/or facilitators. Participants could select more than one option or describe additional barriers/facilitators in free-text responses or select a “none” option. Participants were subsequently asked to state what they felt were the three most important barriers and facilitators, not limited to those from the provided list.
3. *Attitudes (5 questions)*
  Finally, the attitudes section explored participants’ opinions of the importance of intervention fidelity, and views on training needs regarding fidelity. Participants were asked to rate the importance of intervention fidelity on a 5-point scale from 1 (not important) to 5 (very important). Participants were asked to identify any previous training in intervention fidelity (e.g. formal workshops, informal self-directed research) from a list, and could select more than one option or add other types of training. Participants were also asked what types of training they would avail of in future (e.g. workshops, seminars), if any. Finally, participants were given a chance to add any further comments on the survey or topic in general.

### Survey dissemination

The survey was hosted on Google Forms. A total of 297 organisations including healthcare professional groups, research networks and academic institutions from 24 countries were identified. Organisations were contacted via email containing a web link to direct potential participants to the survey. Of these, 42 replied to emails and agreed to disseminate the survey to their members/colleagues. Twitter was also used to disseminate the link to the survey using both general and targeted tweets (e.g. to specific individuals and well-known researchers). The survey was available for completion for a total of 2 weeks and no reminder emails were sent; however, tweets were sent daily over this period.

### Analysis

Descriptive statistics (means, standard deviations) were used to summarise the characteristics of the respondents and levels of knowledge, attitudes and practice of intervention fidelity using SPSS Statistics v24. The survey did not include forced response questions, i.e. participants could proceed to the next question without responding. Questions omitted by participants for individual questions were not imputed, with data generated only from respondents who answered the particular question. The relationship between years of trials-specific experience and (1) self-reported level of knowledge/understanding and (2) attitudes towards fidelity (self-rated importance of fidelity) were explored using Spearman’s correlation. Associations between level of qualification (i.e. doctoral (PhD/DPhil), postgraduate (e.g. MSc.), up to undergraduate (e.g. high school, BSc)), research area and (1) self-reported level of knowledge/understanding and (2) attitudes towards fidelity were explored using one-way between-group analysis of variance (ANOVA). To explore “Research Area”, as participants could select more than one area, three additional grouped categories were created to facilitate analysis. Where participants selected more one category from either medical, allied health professional or nursing/midwifery variables, these were grouped to create “medical and health professionals”. If participants selected both health services research and public health, these were grouped to create “public health and health services research”. Where multiple categories were selected that belonged to more than one of these (i.e. psychology and health services research, or nursing/midwifery and psychology), then they were categorised as “multidisciplinary”. Qualitative data from all four open-ended questions were analysed using a conventional content analysis approach where coding categories are derived directly from the text data, suitable for analysing minimal qualitative data as in our study [31]. Individual responses were summarised by the first author (DMc) according to key emerging concepts or thoughts. These emergent concepts were then grouped and refined into final categories, which were then quantified (i.e. how many responses), with exemplar responses/quotes identified for each (raw data and synthesis are provided in Additional file 2). This synthesis was then double-checked and verified by the corresponding author (ET).

### Ethical approval

Ethical approval for this study was granted by the Galway Clinical Research Ethics Committee. Participants were informed that participation was voluntary and assured of the confidentiality of their responses prior to completing the survey. Brief information on the study aims and funders was provided online at the start of the survey. Participants who subsequently provided electronic consent were invited to continue and complete the survey.

## Results

The online survey received 327 responses. Of these, 62 participants were not eligible as they self-reported no experience of trials of complex healthcare interventions, and data for one participant were excluded as they had experience with pharmaceutical trials only. Therefore, data from 264 participants were included in the analysis. Missing data for individual survey items were minimal (Additional file 3). The mean age of participants was 40.63 (± 11.03) years, with a mean of 11.73 (± 8.67) years of research experience. Participants from 15 countries were included in the analysis, with 60.4% from the Republic of Ireland and England, combined. “Multidisciplinary” was the most represented area of research (43.6% of participants), followed by “medical” (16.2% of participants). Full population demographics are included in Table 1.

**Table 1:** Participant demographics

Variable	Mean ± SD (range)
Age (years)	40.63 ± 11.03
Years of research experience total	11.73 ± 8.67
Years of research experience specific to trials of complex healthcare interventions	7.32 ± 6.75 (18–73)
Variable	*N* (%)
Gender	
Female	203 (76.9)
Male	60 (22.7)
Prefer not to say	1 (0.4)
Country	
Republic of Ireland	91 (34.5)
England	66 (25.9)
Scotland	31 (11.7)
Canada	31 (11.7)
Australia	11 (4.2)
Wales	8 (3.0)
Northern Ireland	6 (2.3)
USA	5 (1.9)
Denmark	4 (1.5)
Norway	3 (1.1)
The Netherlands	2 (0.8)
Switzerland	1 (0.4)
Ethiopia	1 (0.4)
South Africa	1 (0.4)
Italy	1 (0.4)
Prefer not to say	2 (0.8)
Level of qualification	
PhD	127 (48.1)
Masters degree	88 (33.3)
Undergraduate degree	38 (14.4)
MD	9 (3.4)
None	1 (0.4)
Prefer not to say	1 (0.4)
Area of research	
Multidisciplinary (any combination of categories)	116 (43.6)
Medical	43 (16.2)
Allied health professionals	29 (10.9)
Health services research	21 (7.9)
Medical and health professionals (i.e. any combination of Medical, Nursing and Allied health)	16 (6)
Nursing/midwifery	14 (5.3)
Psychology	11 (4.1)
Public health	8 (3)
Public health and health services research	5 (1.9)
Other	3 (1.1)
Level of involvement with trials	
Researcher	174 (65.9)
Principal investigator	78 (29.5)
Research practitioner	52 (19.7)
Trial methodologist	42 (15.9)
Student	37 (14.0)
Practitioner	27 (10.2)
Manager/co-ordinator	23 (8.7)
Epidemiologist	9 (3.4)
Statistician	3 (1.1)
Other	11 (4.2)
Aspect of trials involvement	
Data collection	237 (89.8)
Design/development	201 (76.1)
Reporting	194 (73.5)
Data analysis	177 (67.0)
Delivering the intervention	159 (60.2)
Other	12 (4.5)
Previous training/research in intervention fidelity	
Never received any formal or informal training	137 (51.7)
Informal self-directed research	83 (31.6)
Formal teaching (e.g. lectures, seminars)	24 (9.1)
Formal research (e.g. PhD, MSc)	20 (7.6)
Unsure	1 (0.4)

### Knowledge

**Fig. 1:**
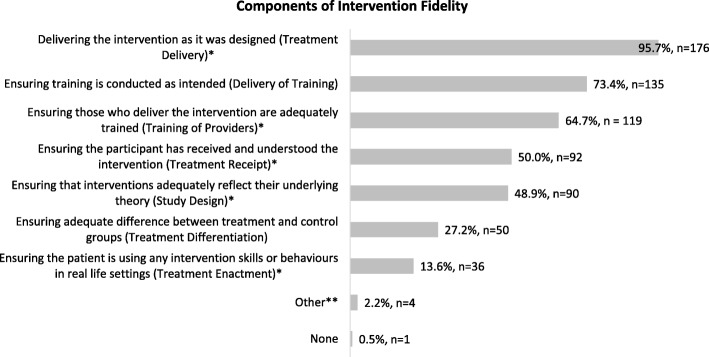
Most commonly endorsed components of intervention fidelity. *NIHBCC domain. **Other = “Determining if fidelity is not delivered if the alternative practice is effective and if so why or why not’ (*n* = 1), ‘Acceptability of intervention to participants and providers” (*n* = 1), “Reproducibility” (*n* = 1), “Ensuring the fidelity criteria are laid out a priori and don’t shift during intervention delivery” (*n* = 1)

Of the 264 respondents, 69.7% (*n* = 184) were familiar with the term “intervention fidelity”. This group were asked to select what they felt were components of intervention fidelity; the vast majority (95.7% (*n* = 176)) indicated “ensuring the intervention is delivered as intended” was a component, reflecting endorsement of the NIHBCC domain “Treatment Delivery”. “Ensuring training given to providers is conducted as intended” was the next most commonly indicated by 73.4% (*n* = 135), while the least frequently endorsed component was the NIHBCC domain of “Treatment Enactment” (13.6% (*n* = 36)). As most participants selected more than one component with a total of 703 responses, the mean number of components reported per participant was 3.82 (SD 1.93). The full list of responses and how participants endorsed the other NIHBCC domains is included in Fig. 1.

Of the participants who were not familiar with the term “intervention fidelity”, 79 responded to a question about synonyms of intervention fidelity. Of these, 70.9% (*n* = 56) were familiar with the term “programme adherence”, 54.4% (*n* = 43) with “implementation adherence” and 49.4% (*n* = 39) with “procedural reliability”: 16 participants (20.3%) responded that they were not familiar with any of these terms. Overall, the mean self-reported knowledge of intervention fidelity for all participants was 5.84 (SD 2.26) on a 10-point scale (prior to the provision of a fidelity definition). There was a significant positive association between years of trials-specific research experience and self-reported knowledge/understanding of fidelity (rho = .260, *n* = 261, *p* < .0005) and between level of qualification and knowledge/understanding (*F* (3, 258) = 10.613, *p* < .0005). People with a PhD or DPhil reported significantly greater knowledge (mean (M) = 6.52, SD = 1.85) than participants with a postgraduate qualification (M = 5.44, SD = 2.47) or those achieving up to an undergraduate qualification (M = 4.50, SD = 2.27). There also was a significant difference in self-reported knowledge/understanding of fidelity based on research area (*F* (9, 28.566) = 4.956, *p* < .0005). Participants in the medical category self-reported significantly lower knowledge (M = 4.37, SD = 2.53) than those in psychology (M = 6.91, SD = 1.04), public health (M = 7.00, SD = 1.60), health services research (M = 6.38, SD = 1.43) and multidisciplinary (M = 6.38, SD = 2.00).

### Practice

**Fig. 2:**
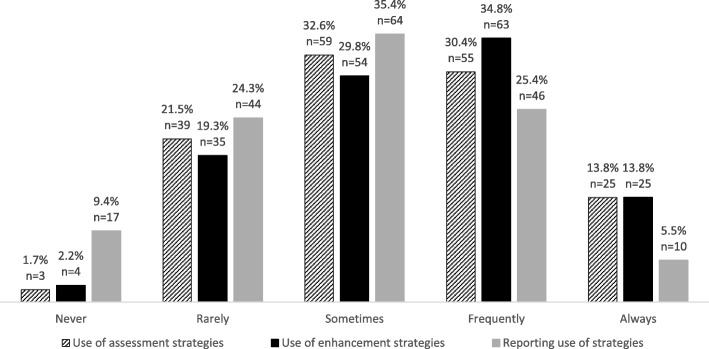
Frequency of use of assessment strategies, enhancement strategies and reporting

The majority (68.9%, *n* = 182) of participants had previous experience of using strategies to assess and/or enhance fidelity in trials of complex interventions. In terms of how frequently these strategies are used, on a scale of 1 (never) to 5 (always), the mean frequency of use of assessment and enhancement strategies were 3.33 (± 1.02) and 3.39 (± 1.02), respectively. 44.2% of participants (*n* = 80) selected “always” or “frequently” using assessment strategies and 48.6% (*n* = 88) selecting the same with respect to enhancement strategies. Using the same scale, the mean frequency of reporting the use of fidelity strategies in publication was 2.93 (± 1.05) with 30.9% (*n* = 56) selecting “always” or “frequently” reporting. The full response to the use of strategies to assess, enhance and report fidelity is presented in Fig. 2.

The mean frequency of reporting the overall results of fidelity assessments (i.e. quantifying the levels of fidelity) was 2.89 (± 1.15) with 29.2% (*n* = 53) selecting “always” or “frequently” reporting. Strategies that participants had previously used to assess (*n* = 181) and enhance (*n* = 182) fidelity are detailed in Table 2. The most frequently used assessment strategies were provider self-report record (*n* = 115), direct observation (*n* = 106) and participant interview (n = 106). The three most frequently used enhancement strategies were training manuals (*n* = 148), reminder checklists (*n* = 137) and treatment manuals or scripted curriculums (*n* = 116).

**Table 2:** Fidelity strategies previously used by participants

Fidelity strategies	Number (%)
Assessment strategies	
Provider self-report record	115 (63.5)
Direct observation	106 (58.6)
Participant interview	106 (58.6)
Provider interview	81 (44.8)
Participant self-report record	73 (40.3)
Audio recording	67 (37)
Participant follow up visits	57 (31.5)
Exit questionnaires	56 (30.9)
Video recording	27 (14.9)
None	1 (0.6)
Other	8 (4.4)
Simulated patients	1 (0.6)
Audit or chart review	2 (1.1)
Web analytics (digital interventions)	3 (1.7)
Blood tests	1 (0.6)
Use of validated fidelity measures	1 (0.6)
Enhancement strategies	
Training manual	148 (81.3)
Treatment manual/scripted curriculum/standard operating procedures	118 (64.8)
Reminder checklists	137 (75.3)
Protocol review group	84 (46.2)
None	4 (2.2)
Other	7 (3.8)
Ongoing support/supervision for providers	2 (1.1)
Observation/audit of providers delivering intervention	3 (1.6)
Colour coding materials for providers	1 (0.5)
Interim analysis	1 (0.5)

**Fig. 3:**
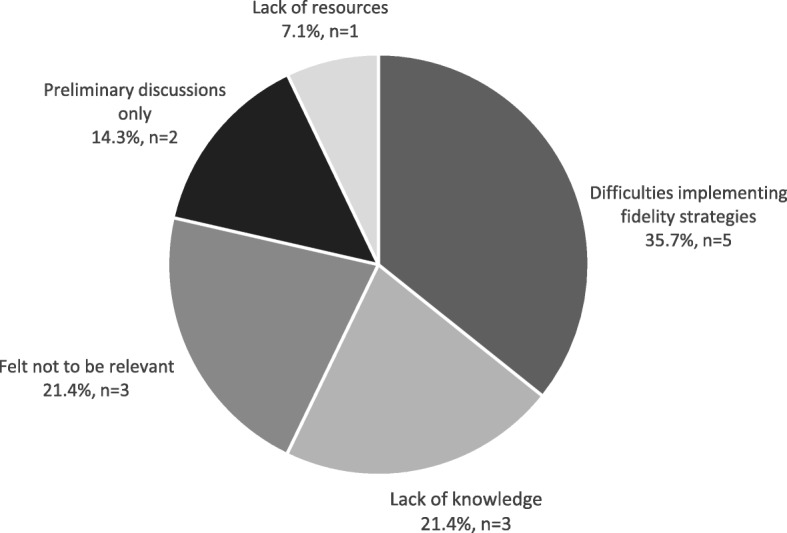
Reasons fidelity strategies were discussed and not used

Of the 82 participants with no previous experience of the use of fidelity strategies, 42.7% (*n* = 35) were unsure whether fidelity strategies were discussed at any stage of trials in which they were involved; 40.2% (*n* = 33) had never discussed fidelity strategies; 17.1% (n = 14) responded that they were discussed but subsequently not used. The main reasons fidelity strategies were reportedly discussed and not used were organised into categories using content analysis (Additional file 2). The most reported category was “difficulty implementing fidelity strategies” (35.7%, *n* = 5). The full response is presented in Fig. 3.

A total of 263 participants responded that they consider or evaluate intervention fidelity during critical appraisal of other trials of complex interventions on an average frequency of 3.07 (SD 1.31), on a five-point scale from 1 (never) to 5 (always).

Of the 258 participants who responded to a question on the use of fidelity frameworks, 73.6% (*n* = 190) had never used a specific fidelity tool or framework. Frameworks or tools that had been used by remaining participants are specified in Table 3. Overall, 15 frameworks were identified by participants. The three most frequently used frameworks were the Updated NIHBCC Treatment Fidelity Framework (*n* = 26) [16], the Conceptual Framework for Implementation Fidelity (n = 26) and the NIHBCC Treatment Fidelity Framework (n = 19) [15].

**Table 3:** Use of fidelity frameworks/tools

	Number (%)
2011 Updated NIHBCC Treatment Fidelity Framework [16]	26 (10.1)
Conceptual Framework for Implementation Fidelity	26 (10.1)
2004 NIHBCC Treatment Fidelity Framework [15]	19 (7.4)
Unsure/do not know	6 (2.3)
Comprehensive Intervention Fidelity Guide [24]	5 (1.9)
Other	15 (5.8)
Medical Research Council Guidance on Process Evaluation of Complex Interventions [40]	3 (1.2)
TIDieR checklist	2 (0.8)
Developed specifically for study	1 (0.4)
Multiple “ad hoc” publications consulted	1 (0.4)
RE-AIM framework [41]	1 (0.4)
Framework/Taxonomy of Implementation [42]	1 (0.4)
Precede-Proceed [43]	1 (0.4)
Conceptual Framework of Implementability [44]	1 (0.4)
Process Evaluation “How-to” Guide [45]	1 (0.4)
BCT Taxonomy v1 [46]	1 (0.4)
Karas and Plankis 2016 [47]	1 (0.4)
Durlak and DuPre 2008 [48]	1 (0.4)
SPIRIT Intervention Fidelity Assessment Tool [49]	1 (0.4)

*NIHBCC* National Institutes of Health Behaviour Change Consortium, *BCT* behaviour change techniques, *TIDieR* Template for intervention description and replication, *RE-AIM* Reach Effectiveness Adoption Implementation Maintenance

A total of 261 participants selected barriers and facilitators to enhancing, addressing or reporting intervention fidelity in trials of complex healthcare interventions, the results of which are included in Tables 4 and 5. There was no difference in the most commonly identified barriers and facilitators for people with an above average understanding/knowledge of fidelity (i.e. mean 5.84) compared to those with a below average score.

**Table 4:** Most frequently identified barriers to enhancing, addressing or reporting intervention fidelity

Barrier	Number (%)
Poor knowledge or understanding	202 (77.4)
Lack of practical guidance	167 (64)
Lack of criteria specifying acceptable levels	164 (62.8)
Inconsistent terminology	148 (56.7)
Time restraints	131 (50.2)
Lack of perceived importance	129 (49.4)
Inconsistent definitions	112 (42.9)
Lack of agreement around appropriate strategies	109 (41.8)
Core components of interventions not sufficiently identified	105 (40.2)
Cost	97 (37.2)
Lack of journal requirement for publication	92 (35.2)
Resistance to monitoring/assessment by providers	82 (31.4)
Resistance to the use of treatment manuals by providers	79 (30.3)
Space limitations for publication	77 (29.5)
None	1 (0.4)
Other^a^	12 (4.6)
“Real-world” complexity and constraints	4 (1.6)
Difficulty quantifying fidelity data	3 (1.2)
Willingness of principal investigator	2 (0.8)
Insufficient teaching/education	1 (0.4)
Rigidity/lack of flexibility may limit patient care	1 (0.4)
Practitioners’ desire for independence	1 (0.4)

^a^Additional barriers identified by participants

**Table 5:** Most frequently identified facilitators to enhancing, addressing or reporting intervention fidelity

Facilitator	Number (%)
Knowledge of how to assess or enhance	209 (80.1)
Availability of validated tools or checklists	202 (77.4)
Availability of practical guidance	180 (69)
Clear understanding of the definition	168 (64.4)
Perceived importance by researchers	164 (62.8)
Funding or monetary resources	136 (52.1)
Perceived importance by academic journals	132 (50.6)
Accessibility of methodologists or people with specific fidelity expertise	123 (47.1)
Availability of reporting criteria	121 (46.4)
Time	114 (43.7)
Priority given by journals	84 (32.2)
Do not know	2 (0.8)
Other^a^	7 (2.7)
Perceived importance by funders	2 (0.8)
Perceived importance by providers	2 (0.8)
Perceived importance by principal investigators	1 (0.4)
Translation from research to real world setting	1 (0.4)
Training	1 (0.4)

^a^Additional facilitators identified by participants

When asked to rate the most important barriers and facilitators, 170 participants ranked time constraints (*n* = 71), lack of knowledge/understanding (*n* = 64) and cost (*n* = 59) as the top barriers. The top three facilitators as ranked by 161 respondents were: availability of validated tools or checklists (*n* = 61), good knowledge of how to assess or enhance fidelity (*n* = 54), and availability of funding (*n* = 48).

### Attitudes

The majority of participants either rated fidelity as “Very Important” (57.1%, *n* = 149) or “Important” (33%, *n* = 86), with 260 participants rating the importance of intervention fidelity in trials of complex interventions as high (mean 4.47 ± 0.67 on a scale of 1–5). There was no association between years of trials-specific research experience and attitudes (i.e. perceived importance) towards fidelity (rho = .093 *n* = 260, *p* = .137); however, there was a significant difference in attitudes based on qualification level (*F* (3, 30.45) = 4.808, *p* = .007). Participants with a PhD or DPhil perceived fidelity as more important (M = 4.62, SD = 0.61) than people with a postgraduate qualification (M = 4.33, SD = 0.84) or those achieving up to an undergraduate qualification (M = 4.13, SD = 0.97). There was also a significant difference in attitudes towards fidelity based on research area (*F* (9, 28.244) = 2.93, *p* = .014). Participants in the combined group “medical and health professionals” (M = 3.56, SD = 1.26) reported perceiving fidelity as less important than allied health professionals (M = 4.69, SD = 0.47), psychology (M = 4.67, SD = 0.50), public health (M = 4.88, SD = 0.35), nursing/midwifery (M = 4.43, SD = 0.65), health services research (M4.62, SD = 0.59) and multidisciplinary (M = 4.53, SD = 0.63). No other significant differences were observed. In terms of training, 89.7% (*n* = 236) responded that they would welcome training if it was available, with workshops (*n* = 177), webinars (*n* = 163) and online courses (*n* = 163) rated as the most popular, followed by seminars (*n* = 119), lectures (*n* = 97) and conference presentations (*n* = 1). Reasons provided by the remaining 10.3% (*n* = 27) for not wanting further training were cited as a lack of relevance to work (*n* = 12), having sufficient understanding or access to online information already (*n* = 12), time constraints (*n* = 7), being retired (*n* = 2), having other priorities (*n* = 1), or  not considering it important (*n* = 1).  

Thirty-two participants provided additional comments related to attitudes about intervention fidelity (provided in Additional file 2). Results of content analysis indicated that these mostly focused on the importance of intervention fidelity, practicalities, issues regarding terminology/definitions and the need for further training. Sample quotes from this content analysis are provided in Table 6.

**Table 6:** Additional comments regarding intervention fidelity

Theme identified (number of participants)	Sample quotes
Importance of intervention fidelity (*n* = 18)	“This is an important and interesting aspect to clinical trials …”
	“… an important issue, may undermine the lack of findings from some trials”
	“I think fidelity is central to the findings of any complex trial. I am a strong advocate …”
	“… an incredibly important area of research”
Practicalities (*n* = 7)	“It is difficult to assess treatment fidelity as the resource needed to do this properly is quite significant …”
	“Pragmatism has to trump strict adherence/fidelity in complex healthcare interventions …”
	“Ideally, complex intervention clinical trials should be preceded by a feasibility study …”
Terminology/definitions (*n* = 3)	“… it’s not a term that I was familiar with pre-survey”
	“… something that does not appear to have a clear definition … I cannot be sure that I have answered your questions accurately”
Further training (*n* = 3)	“… would gladly apply teaching regarding this topic”
	“Re training: I think it is vital for young researchers - wish it had been around when I was starting out”

## Discussion

This is the first survey of intervention fidelity amongst those involved in the development, conduct and evaluation of trials of complex healthcare interventions. The survey provides insight into past and current intervention fidelity practices, knowledge and attitudes. Findings highlight an awareness of intervention fidelity and its importance amongst those with experience of trials of complex interventions, but also a lack of knowledge and need for further training and education in intervention fidelity. The findings further demonstrated a discrepancy between use and reporting of fidelity strategies, and a need for practical guidance and information to improve research in this area.

The findings of this survey clearly show that a lack of knowledge and understanding is one of the key challenges to intervention fidelity. Over 90% of the participants in this study were aware of intervention fidelity or a similar term; however, self-reported understanding was rated as only slightly above average. Although participants with more years of trial-specific experience reported better knowledge, issues relating to knowledge and understanding were the most frequently reported barriers and facilitators of intervention fidelity by participants regardless of their self-reported understanding. These issues were also ranked as the *most important* barriers and facilitators. Perepletchikova et al. and Cochrane et al. similarly found lack of knowledge to be a barrier to “treatment integrity” procedures amongst psychotherapy researchers [27] and teachers involved in intervention provision, respectively [28]. However, the lack of specific theory and guidance and resource issues such as time and cost were found to be stronger barriers for psychotherapy research [27]. As intervention fidelity emerged earlier in psychotherapy literature [11, 21, 32], this may account for this slight difference in findings, reflecting the differing survey populations. Nonetheless, barriers relating to the lack of practical guidance and resources were also found to be important in our survey. Again, in keeping with findings by Perepletchikova et al. and Cochrane et al. [27, 28], participants in our survey felt that intervention fidelity was highly important, regardless of their years of trials experience, with zero participants designating intervention fidelity as “of little or no importance”. Although participants with a doctoral level of qualification had better self-reported understanding and rated fidelity as more important than those at an undergraduate or postgraduate level, this may reflect the deeper level of reflection and engagement with research that is typically expected at doctoral level studies. Nonetheless, with the majority of participants rating it as important but reporting poor knowledge and key barriers relating to knowledge and understanding, it is clear that additional training and education in intervention fidelity is both needed and wanted. This is further evidenced by the fact that almost 90% of participants reported they would avail of such training. As both knowledge and attitudes towards fidelity seemed to be somewhat lower in areas involving medical research, developing training that clearly highlights the relevance of intervention fidelity for all applicable research areas from the outset and utilises appropriate examples from medical research as well as other areas, may be warranted.

The variability in participants’ perceptions of the components of fidelity provides evidence that conceptualisation of intervention fidelity often varies between researchers. This finding is perhaps unsurprising given the lack of consensus in the literature on fidelity definitions, terminology and conceptualisations [4]; however, this discrepancy makes it difficult to move the science forward [19]. That “treatment delivery” was the most frequently endorsed component of intervention fidelity is perhaps unsurprising, as delivering the treatment as intended represents where intervention fidelity literature originated [15, 21], and is the most frequently measured or reported domain of intervention fidelity [8, 14, 24]. A small number of participants in this study (*n* = 20) felt treatment delivery was the only component of fidelity, perhaps representing a somewhat limited view of intervention fidelity. However, as previously mentioned, intervention fidelity has essentially evolved into a multi-component, multidimensional concept, enabling a more comprehensive understanding of the intervention process [15]; this likely adds to the confusion evidenced by the participants in this survey. However, it may also be the case that participants were aware of the different components provided, but may not have agreed with their inclusion within the concept of intervention fidelity. For example, the most infrequently selected fidelity component was enactment, potentially in line with previous arguments that treatment enactment is a measure of treatment effectiveness and not fidelity [24]. This finding was also echoed in two recent systematic reviews of fidelity within complex interventions by O’Shea et al. [8] and Lambert et al., [14] who both found treatment enactment to be infrequently measured, carried out in only 10 out of 65 and 8 out of 21 included studies, respectively. Overall, the variability in selected components of fidelity, in addition to participants’ low self-reported understanding score, suggest an overall lack of clarity in this population on what intervention fidelity is and what it encompasses. As the majority of participants felt that a clear understanding of the definition was a facilitator to intervention fidelity in this area, it would appear that a universally agreed upon definition and conceptualisation of intervention fidelity is essential to improving knowledge, understanding and practice of fidelity in trials of complex interventions.

The findings of this survey also highlight disparities between participants’ use of fidelity strategies and their subsequent reporting of these strategies in publication. A commonly cited limitation of many systematic reviews that have evaluated fidelity practices is that it is often unclear whether intervention fidelity was poorly addressed or just poorly reported in published intervention studies [4, 8, 10]. This survey provides evidence that even when strategies to enhance and assess intervention fidelity are utilised within trials of complex interventions, they are not always subsequently reported. In our survey, participants identified 14 ways to assess fidelity and 8 ways to enhance fidelity in trials. Adequate reporting of any such methods that are used facilitates optimal selection of strategies for researchers involved in future trials, and provides more information on how to choose these more appropriately [4, 17]. Although reporting of intervention fidelity may be influenced by issues such as space restrictions and lack of journal reporting requirements [27], these were not the most common nor the most important perceived barriers or facilitators identified in this study. Therefore, although we support previous recommendations for journals to request fidelity details and provide space for reporting [4], it may be more of a research priority to facilitate a more standardised approach to reporting fidelity. This could be done through the development of specific fidelity reporting criteria, or expansion of existing criteria such as items 11 and 12 of the Template for intervention description and replication (TIDiER) guidelines [33], so that researchers are equipped with more accurate and up-to-date knowledge of what to report and how.

The importance of tools or checklists and practical guidance recurred throughout the survey data, with the availability of these items featuring amongst the most important and most frequently reported facilitators. Despite this finding, the majority of participants had never used a validated framework or tool such as the NIHBCC fidelity framework [9] or the Conceptual Framework for Implementation Fidelity [2], which were the most commonly identified frameworks by those who had used one. Previous research has similarly found that fidelity frameworks are underused in behaviour change interventions, despite their availability and importance [8, 13]. Although O’Shea and colleagues suggested that their underuse may have been due to lack of resources [8], the findings of this survey posit that a lack of awareness of such tools may also be an issue. Our findings also suggest that suboptimal use of such frameworks may also be due to a lack of usability or practicality issues, an issue highlighted recently by Walton et al., who found that only 26% of health behaviour change interventions focused on acceptability and practicality of fidelity assessment measures [13]. Previous studies that have used the NIHBCC framework have also highlighted issues in relation to ambiguity of components [34], or a lack of practical guidance in terms of how to incorporate actual fidelity scores [35]. Moreover, neither the NIHBCC nor the Conceptual Framework for Implementation Fidelity framework provide sufficient guidance on how to balance fidelity with adaptation. This is an issue of vital importance within this area [36–38], briefly alluded to by one participant in the additional comments regarding the need for pragmatism over strict fidelity. These frameworks also do not discuss weighting of components, making it difficult for researchers to determine which intervention fidelity components to prioritise if limited for time or resources. With time and funding featuring amongst the most important barriers and facilitators, our findings echo previous research, which identifies time, labour and cost to be strong barriers to intervention fidelity [27, 39]. As such, future research should focus on developing practical guidance and/or improving existing frameworks to address intervention fidelity in trials in a way that considers the issue of adaptation and is mindful of time and cost issues for researchers.

### Study limitations

In this survey, barriers and facilitators to enhancing, assessing and reporting intervention fidelity were explored concurrently. However, barriers may be experienced differently for these aspects, for example, time restraints may be a bigger barrier to using assessment strategies (i.e. conducting direct observations) than to enhancing fidelity (i.e. using a treatment manual). Future research could therefore explore the barriers and facilitators more specifically to each aspect individually. Due to limited resources, psychometric testing was not carried out on survey questions. Moreover, survey questions were predominantly informed by the NIHBCC conceptualisation of intervention fidelity, which may have influenced findings. However, other predominant fidelity literature and conceptualisations were utilised in the questionnaire development and questions were piloted to minimise the potential for bias. Additionally, the majority of respondents were based in Ireland and the UK, potentially reflecting the opportunistic nature of the sample and the authors’ locations, and may influence the generalisability of study findings. However, attempts were made to disseminate the survey as broadly as possible, and a total of 15 countries were represented in this survey. Additionally, the response rate from organisations contacted was low (14%), which may also impact the generalisability of our findings. This low response rate perhaps demonstrates low interest in the important issue of intervention fidelity amongst the wider complex healthcare intervention research community. However, no reminder emails were sent and the majority of these organisations were contacted using generic public email addresses where the initial contact point may not have been a researcher or had much involvement with complex healthcare intervention research, therefore the survey link or information about the study may not have reached the relevant parties. It must also be acknowledged that those who participated in this survey may have been more interested in intervention fidelity from the outset, i.e. a self-selection bias meaning that those with the most interest and awareness of fidelity were most likely to complete the survey. Nonetheless, this means our findings likely represent a best-case scenario and the lack of awareness and use of fidelity in the wider complex healthcare intervention literature is actually higher. However, if this is the case, it further emphasises the recommendations from this study, highlighting the need for further research and training to increase awareness and understanding of this important issue. Furthermore, participants in this survey had variable levels of education and experience specific to intervention fidelity as well as representing a broad range of trial involvement and multidisciplinary research areas, which enhances the generalisability of the study findings.

## Conclusions

Despite good awareness of intervention fidelity and its importance, poor knowledge and understanding appears to be a substantial limitation in how intervention fidelity is being addressed in trials of complex healthcare interventions. Clarification and universal agreement around the terminology, definition and components of intervention fidelity would facilitate better understanding of the concept. Participants identified a need for training in this area, and felt that practical guidance on how to assess, enhance or report fidelity in trials of complex interventions is lacking. Discrepancies between participants’ previous use of fidelity strategies and subsequent reporting highlights the issue of inadequate intervention fidelity reporting, identifying a further area for future development.

## Additional files

Additional file 1: Survey – “Surveying knowledge, practice and attitudes towards intervention fidelity within trials of complex healthcare interventions”. (PDF 136 kb)

Additional file 2: Open-ended questions from survey. (XLSX 38 kb)

Additional file 3: Dataset: anonymized raw data. (XLS 419 kb)

## Abbreviations

BCT: Behaviour change techniques
MRC: Medical Research Council
NIHBCC: National Institute of Health, Behaviour Change Consortium
RE-AIM: Reach effectiveness adoption implementation maintenance
TIDieR: Template for intervention description and replication
