# Supplementary material for: Surveying knowledge, practice and attitudes towards intervention fidelity within trials of complex healthcare interventions
Source: Trials. 2018 Sep 19;19:504. doi: 10.1186/s13063-018-2838-6 (PMC6147031; doi:10.1186/s13063-018-2838-6)
Supplement: Supplementary file 1 — Survey – “Surveying knowledge, practice and attitudes towards intervention fidelity within trials of complex healthcare interventions”. (PDF 136 kb) [file 13063_2018_2838_MOESM1_ESM.pdf]

Surveying knowledge, practice and attitudes toward intervention fidelity within trials of complex healthcare interventions

This study has been funded by the Irish Health Research Board Trials Methodology Research Network summer studentship awards, and is being led by researchers from the Health Behaviour Change Research Group, National University of Ireland Galway, Dr Elaine Toomey (PI) and Mr Daragh McGee (HRB-TMRN summer student). Ethical approval for this study has been granted by the Galway Clinical Research Ethics Committee.

* Required

Introduction

This survey will explore the knowledge, practice and attitudes that researchers, practitioners and others with experience of clinical trials have toward intervention fidelity in trials of complex interventions in healthcare. This is to inform future research and training needs in relation to intervention fidelity practices.

The Medical Research Council describes complex interventions as having several interacting components, e.g. number of groups targeted by the intervention, number of outcomes (Craig et al, 2008). A trial is a research study that prospectively assigns human participants or groups of humans to one or more health-related interventions to evaluate the effects on health outcomes. For this study we are recruiting those with experience of trials of any complex health intervention (this excludes pharmacological/drug trials).

1. **Responses to this survey are voluntary, confidential and will not involve any personal identifying material. By participating, you are certifying that you are over the age of 18 and agreeing to allow us to anonymously publish the research ideas that you help us to identify.** *

   Check all that apply.

   ☐ I agree

2. **Are you or have you been directly involved (e.g. design/development, intervention delivery, data collection, evaluation) with trials of complex interventions in healthcare? (Experience as a study participant/subject only is not sufficient for the purposes of this survey)** *

   Mark only one oval.

   ◯ Yes

   ◯ No *Stop filling out this form.*

Background

3. **What age are you?**

   ____

4. **What is your gender?**
Mark only one oval.

- Male
- Female
- Other: ____________

5. **What country do you work in?**
Mark only one oval.

- Republic of Ireland
- Northern Ireland
- Scotland
- Wales
- England
- Other: ____________

6. **What is the highest qualification you have completed?**
Mark only one oval.

- PhD
- Masters degree
- Undergraduate degree
- MD
- Other: ____________

7. **What area of research are you involved with? (Tick all that apply)**
Check all that apply.

- [ ] Medical
- [ ] Nursing/midwifery
- [ ] Allied health professionals
- [ ] Psychology
- [ ] Public health
- [ ] Health services research
- [ ] Other: ____________

8. **What best describes your level of involvement with trials of complex interventions? (Tick all that apply)**
Check all that apply.

- [ ] Researcher
- [ ] Principal investigator
- [ ] Trial methodologist (someone who specializes in the methods of how trials are designed, run, analyzed and reported)
- [ ] Statistician
- [ ] Student
- [ ] Epidemiologist
- [ ] Research practitioner
- [ ] Practitioner
- [ ] Other: ____________________

9. **What aspect(s) of trials of complex interventions have you been involved with? (Tick all that apply)**
Check all that apply.

- [ ] Design/development
- [ ] Delivering the intervention
- [ ] Data collection
- [ ] Data analysis
- [ ] Reporting
- [ ] Other: ____________________

10. **How many years of research experience do you have in total?**

____

11. **How many years of research experience do you have specific to trials of complex interventions in healthcare?**

____

Knowledge

12. **Are you familiar with the term intervention fidelity?**
Mark only one oval.

- ( ) Yes *Skip to question 13.*
- ( ) No *Skip to question 14.*

Yes, I am familiar with intervention fidelity

13. **Which of the following do you think are components of intervention fidelity? (Tick all that apply)**
Check all that apply.

- [ ] Ensuring that interventions adequately reflect their underlying theory (e.g. Theory of Planned Behaviour) or hypothesised mechanisms of action (e.g. using mediation analysis)
- [ ] Ensuring adequate difference between the treatment and control groups (i.e. treatment differentiation)
- [ ] Ensuring those who deliver the intervention (e.g. doctors, therapists, allied health professionals) are adequately trained
- [ ] Ensuring that any training given to intervention providers is conducted as intended
- [ ] Delivering the intervention to the participants or patients as it was designed
- [ ] Ensuring that the participant or patient has received (e.g. attended) and understood the intervention
- [ ] Ensuring that the participant is using any intervention skills or behaviours in real life
- [ ] None of the above
- [ ] Other: ____________________

Skip to question 15.

No, I am not familiar with intervention fidelity

14. **There are many terms that have previously been used synonymously with intervention fidelity; are you familiar with any of these terms? (Tick all that apply)**
Check all that apply.

- [ ] Treatment fidelity
- [ ] Implementation fidelity/fidelity of implementation
- [ ] Implementation adherence
- [ ] Programme adherence
- [ ] Theoretical fidelity
- [ ] Strategy fidelity
- [ ] Fidelity monitoring
- [ ] Programme fidelity
- [ ] Implementation integrity
- [ ] Treatment integrity
- [ ] Procedural reliability
- [ ] I am not familiar with any of these terms

Knowledge

15. **How well would you describe your understanding of intervention fidelity?**
Mark only one oval.

	1	2	3	4	5	6	7	8	9	10	
Poor	◯	◯	◯	◯	◯	◯	◯	◯	◯	◯	Excellent

Definition

Intervention fidelity has been described in the literature as the degree to which programmes are implemented as intended by the programme developers (Carroll et al, 2007); in other words, how true

the intervention is being practiced or delivered in relation to its original design.

Practice

16. **In your experience of trials of complex interventions, was intervention fidelity ever assessed (i.e. the use of strategies to assess fidelity to the intervention) or enhanced (i.e. the use of strategies to improve fidelity to the intervention)?**
Mark only one oval.

- ( ) Yes *Skip to question 17.*
- ( ) No *Skip to question 23.*

Yes, I have been involved in trials where intervention fidelity methods were used

17. **Were any of these methods used to specifically assess intervention fidelity? (Tick all that apply)**
Check all that apply.

- [ ] Direct observation
- [ ] Audio-recording
- [ ] Video-recording
- [ ] Provider self-report record
- [ ] Provider interview (in person, or by phone)
- [ ] Participant self-report record
- [ ] Participant follow-up visits
- [ ] Participant interview (in person, or by phone)
- [ ] Exit questionnaires
- [ ] None
- [ ] Other: ______________________

18. **In your experience of trials of complex interventions, how often have strategies to assess intervention fidelity been used (on average)?**
Mark only one oval.

	1	2	3	4	5	
Never	( )	( )	( )	( )	( )	Always

19. **Were any of these strategies used to enhance intervention fidelity? (Tick all that apply)**
Check all that apply.

- [ ] Protocol review group (i.e. an expert committee to review and amend clinical protocols before implementation)
- [ ] Treatment manual or scripted curriculum
- [ ] Training manual (to standardize training given to those delivering the intervention)
- [ ] Reminder checklists (used before delivery by those delivering the intervention)
- [ ] None
- [ ] Other: ______________________

20. **In your experience of trials of complex interventions, how often have strategies to enhance intervention fidelity been used (on average)?**
Mark only one oval.

	1	2	3	4	5	
Never	⬭	⬭	⬭	⬭	⬭	Always

21. **In your experience of trials of complex interventions, how well has the use of these intervention fidelity methods been reported?**
Mark only one oval.

	1	2	3	4	5	
Poor	⬭	⬭	⬭	⬭	⬭	Excellent

22. **In your experience of trials of complex interventions, how often has the actual result of any fidelity assessments (if applicable) been reported?**
Mark only one oval.

	1	2	3	4	5	
Never	⬭	⬭	⬭	⬭	⬭	Always

Skip to question 25.

No, I have not been involved in trials where intervention fidelity methods were used

23. **Were methods of assessing or enhancing intervention fidelity discussed at any stage of the trial?**
Mark only one oval.

- ⬭ Yes
- ⬭ No
- ⬭ Not sure

24. **If you answered yes above, why were they not used?**

____

Practice

25. **In your experience of trials of complex interventions, have you ever used any of the following frameworks to inform how fidelity might be enhanced, assessed or reported)? (Tick all that apply)**

Check all that apply.

- [ ] National Institute of Health Behaviour Change Consortium Treatment Fidelity Framework tool (Borrelli et al, 2005)
- [ ] Updated National Institute of Health Behaviour Change Consortium Treatment Fidelity Framework tool (Borrelli, 2011)
- [ ] Comprehensive Intervention Fidelity Guide (Gearing et al, 2010)
- [ ] Conceptual Framework for Implementation Fidelity (Carroll et al, 2007)
- [ ] None
- [ ] Other: ____________________

26. **How often do you evaluate/consider intervention fidelity practices during critical appraisal of other trials of complex interventions?**

Mark only one oval.

	1	2	3	4	5	
Never	◯	◯	◯	◯	◯	Always

27. **Which, if any, of the following do you think may be barriers to enhancing, addressing or reporting intervention fidelity in trials of complex healthcare interventions? (Tick all that apply)**

Check all that apply.

- [ ] Poor knowledge or understanding of intervention fidelity
- [ ] Inconsistent terminology surrounding intervention fidelity (i.e. too many synonyms)
- [ ] Ambiguity and lack of consistency surrounding the definition of intervention fidelity (i.e. too many definitions)
- [ ] Lack of criteria specifying acceptable levels of intervention fidelity
- [ ] Lack of practical guidance about intervention fidelity
- [ ] Lack of agreement regarding the most appropriate strategies of enhancement/assessment
- [ ] Lack of perceived importance of intervention fidelity
- [ ] Resistance/perceived resistance from those who deliver the intervention regarding the use of treatment manual (e.g. limiting treatment flexibility)
- [ ] Resistance/perceived resistance from those who deliver the intervention around monitoring/assessment
- [ ] Core components of interventions not sufficiently identified
- [ ] Space limitations for journal publication
- [ ] Lack of journal requirement for publication
- [ ] Cost
- [ ] Time restraints
- [ ] None
- [ ] Other: ____________________

28. **Please list what you feel are the three most important barriers to assessing, enhancing addressing or reporting intervention fidelity in trials of complex healthcare interventions.**

____
____
____
____
____

29. **Which, if any, of the following do you think may be enablers/facilitators to enhancing, addressing or reporting intervention fidelity in trials of complex healthcare interventions? (Tick all that apply)**

Check all that apply.

- [ ] Clear understanding of the definition of intervention fidelity
- [ ] Good knowledge of how to assess or enhance it
- [ ] Availability of validated tools or checklists for assessment or enhancement
- [ ] Availability of practical guidance on strategies and how to adapt them to individual trials
- [ ] Perceived importance by researchers
- [ ] Perceived importance by academic journals
- [ ] Availability of reporting criteria specific to intervention fidelity
- [ ] Space allowances/reporting requirements within academic journals
- [ ] Accessibility of methodologists or people with skills to implement intervention fidelity strategies
- [ ] Funding or monetary resources
- [ ] Time
- [ ] Other: _______________

30. **Please list what you feel are the three most important enablers/facilitators to assessing, enhancing addressing or reporting intervention fidelity in trials of complex healthcare interventions.**

____
____
____
____
____

Attitudes

31. **Based upon your understanding of intervention fidelity, how important do you consider it to be in trials of complex interventions in healthcare?**
Mark only one oval.

- Very important
- Important
- Undecided
- Of little importance
- Not important
- Not relevant to my area

32. **Have you ever received any training/completed specific research into intervention fidelity? (Tick all that apply)**
Mark only one oval.

- Never
- Received formal teaching (e.g. attended workshops, seminars, lectures)
- Informal self directed research (e.g. self-directed reading)
- Formal research (e.g. PhD, MSc in this or related area)
- Other: ______________________

33. **Would you avail of training in intervention fidelity if it was available?**
Mark only one oval.

- Yes *Skip to question 34.*
- No *Skip to question 35.*

Yes, I would avail of training

34. **What type of training would you attend? (Tick all that apply)**
Check all that apply.

- [ ] Workshops
- [ ] Webinars
- [ ] Seminars
- [ ] Lectures
- [ ] Online courses
- [ ] Other: ______________________

Skip to question 36.

No, I would not avail of training

35. **Why not? (Tick all that apply)**

Check all that apply.

- [ ] I don't consider it relevant to my work
- [ ] I wouldn't have the time
- [ ] I have a good understanding of this topic already
- [ ] I don't consider it important
- [ ] Other: ____________________

Attitudes

36. **Do you have any comments you wish to add?**

____

____

____

____

____

Thank you!

If you have any questions or concerns, or would like to receive updates about the outcomes of this research please contact Dr Elaine Toomey by email at elaine.toomey@nuigalway.ie.

Powered by
Google Forms
